# Identifying and predicting the pathogenic effects of a novel variant inducing severe early onset MMA: a bioinformatics approach

**DOI:** 10.1186/s41065-023-00281-0

**Published:** 2023-05-29

**Authors:** Fereshteh Maryami, Elham Rismani, Elham Davoudi-Dehaghani, Nasrin Khalesi, Fatemeh Zafarghandi Motlagh, Alireza Kordafshari, Saeed Talebi, Hamzeh Rahimi, Sirous Zeinali

**Affiliations:** 1 Department of Molecular Medicine, Biotechnology Research Center, Pasteur Institute of Iran, Pasteur St., Tehran, Iran; 2 Department of Pediatrics and Neonatal Intensive Care Unit, Ali-Asghar Children’s Hospital, Iran University of Medical Sciences, Vahid Dastgerdi Street, Modarres Highway, Tehran, Iran; 3 Medical Genetics Lab, Kawsar Human Genetics Research Center, No. 41 Majlesi St., ValiAsr St., Tehran, Iran; 4 Department of Medical Genetics and Molecular Biology, Faculty of Medicine, Iran University of Medical Sciences (IUMS), Tehran, Iran; 5 Department of Medical Genetics, Ali-Asghar Children’s Hospital, Iran University of Medical Sciences, Tehran, Iran; 6 Present address: Texas Biomedical Research Center, San Antonio, USA

**Keywords:** Methylmalonic acidemia, Whole-exome sequencing, *MMAB*, Corrinoid adenosyltransferase, Homology modeling, Bioinformatics

## Abstract

**Background:**

Methylmalonic acidemia (MMA) is a rare metabolic disorder resulting from functional defects in methylmalonyl-CoA mutase. Mutations in the *MMAB* gene are responsible for the *cblB* type of vitamin B12-responsive MMA.

**Results:**

This study used Whole-exome sequencing (WES), Sanger sequencing, linkage analysis, and in-silico evaluation of the variants’ effect on protein structure and function to confirm their pathogenicity in a 2-day-old neonate presenting an early-onset metabolic crisis and death. WES revealed a homozygous missense variant on chromosome 12, the NM_052845.4 (*MMAB*):c.557G > A, p.Arg186Gln, in exon 7, a highly conserved and hot spot region for pathogenic variants. After being confirmed by Sanger sequencing, the wild-type and mutant proteins’ structure and function were modeled and examined using in-silico bioinformatics tools and compared to the variant NM_052845.4 (*MMAB*):c.556C > T, p.Arg186Trp, a known pathogenic variant at the same position. Comprehensive bioinformatics analysis showed a significant reduction in the stability of variants and changes in protein–protein and ligand–protein interactions. Interestingly, the variant c.557G > A, p.Arg186Gln depicted more variations in the secondary structure and less binding to the ATP and B12 ligands compared to the c.556C > T, p.Arg186Trp, the known pathogenic variant.

**Conclusion:**

This study succeeded in expanding the variant spectra of the *MMAB*, forasmuch as the variant c.557G > A, p.Arg186Gln is suggested as a pathogenic variant and the cause of severe MMA and neonatal death. These results benefit the prenatal diagnosis of MMA in the subsequent pregnancies and carrier screening of the family members. Furthermore, as an auxiliary technique, homology modeling and protein structure and function evaluations could provide geneticists with a more accurate interpretation of variants’ pathogenicity.

**Graphical Abstract:**

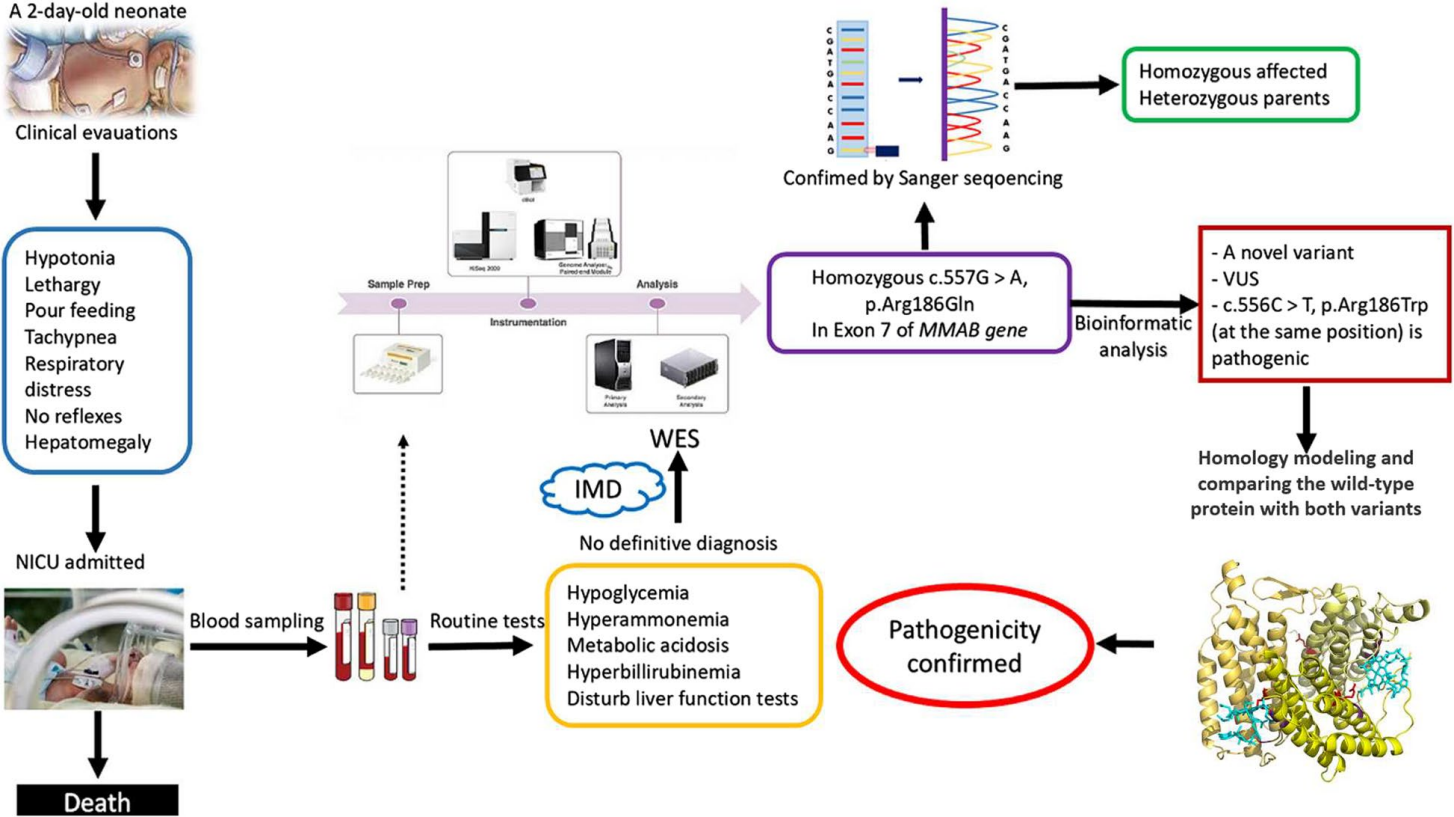

## Background

Methylmalonic acidemia (MMA) is a rare metabolic disorder with an incidence rate of less than 1:100,000 in North America, Europe, and Asia–Pacific regions, however, the rate is higher in the Middle East, North Africa, and Japan [1]. MMA results from functional defects in methylmalonyl-CoA mutase (MCM, EC 5.4.99.2) [2], a mitochondrial enzyme involved in the catabolism of branched-chain amino acids, odd-chain fatty acids, and cholesterol. AdoCbl, produced from cob(I)alamin by ATP:Cobalamin adenosyltransferase (ATR), encoded by *MMAB* gene [3, 4], is directly transferred to the *mut* complementation group (MCM) to play its catalytic role. Subsequently, the *cblA*, encoded by the *MMAA* gene, acts as a gatekeeper to ensure that the AdoCbl is accurately accepted and retained by the MCM [5–9].

Biallelic pathogenic variants in *MMUT, MMAA*, *MMAB*, *MCEE,* and *MMADHC* are responsible for isolated MMA, among which mutations in *MMUT* are the most common cause. Besides, *MMU*T and *MMAB* variants could express the most severe clinical presentations in the neonatal period, including lethargy, vomiting, hypotonia, respiratory distress, severe ketoacidosis, hyperammonemia, and may result in metabolic encephalopathy and death within the first month of life (https://www.ncbi.nlm.nih.gov/books/NBK1231/#); however, the milder chronic form of the disease, with less neurological impairment, have also been reported [10].

*MMAB* gene, with 19,790 bp length, is located on chromosome 12, producing a total of four different transcript variants, all of which encode a 250-residue-length protein named Corrinoid adenosyltransferase (ATP:Cob(I)alamin adenosyltransferase) (ATR) (EC.2.5.1.17). ATR belongs to the PduO family of cobalamin adenosyltransferase. Like the Pduo enzymes, ATR has a homotrimeric structure, each subunit comprising five helix bundles with 32–40% sequence identity. Based on the current knowledge about the protein structure, the active site seems to lie in a cleft at the subunits interface [9, 10].

Identification of PduO structure and modeling of the variant proteins shed light on the molecular pathogenicity of the genetic variations. To date, 48 different pathogenic variants have been reported in *cblB*-MMA patients; 26 out of 48 are missense/nonsense variants (HGMD® Professional Release 2021.4) (2022). The pathogenicity of these variants is considerably linked to the impact of the variant on protein structure and function. Thus, some amino acid codons are relatively conserved, two of which, Arg190 and Arg186, have been modeled on the crystal structure of human ATR and ATP complexes. As a result, Arg190 was proposed to be the ATP binding site, while Arg186 was supposed to interact with cobalamin [3]. Recently, more variants have been identified through high throughput technologies such as whole-exome sequencing (WES). However, homology modeling and variant protein evaluations would gain better insight into the protein structure and function.

In this study, WES was utilized to assess the genetic variation underlying the disease and death of a 2-day-old neonate who had been admitted to the Neonatal Intensive Care Unit (NICU) for severe hyperammonemia, metabolic acidosis, and encephalopathy. WES data analysis revealed a homozygous variant, NM_052845.4 (*MMAB*):c.557G > A, p.Arg186Gln, in exon 7 of the gene, suggesting isolated methylmalonic acidemia, *cblB* type. Through a comprehensive bioinformatics approach, the effect of the variant on protein structure and function was evaluated and compared to the NM_052845.4 (*MMAB*):c.556C > T, p.Arg186Trp, the known pathogenic variant at the same position [5], reported in ClinVar (https://www.ncbi.nlm.nih.gov/clinvar/) and UniProt (https://www.uniprot.org/) datasets. Finally, the variant’s pathogenicity was confirmed by in-silico analysis of both variants’ effect on protein structure and function and categorized regarding the American College of Medical Genetics and Genomics (ACMG) guideline.

## Results

### Clinical features

**Fig. 1:**
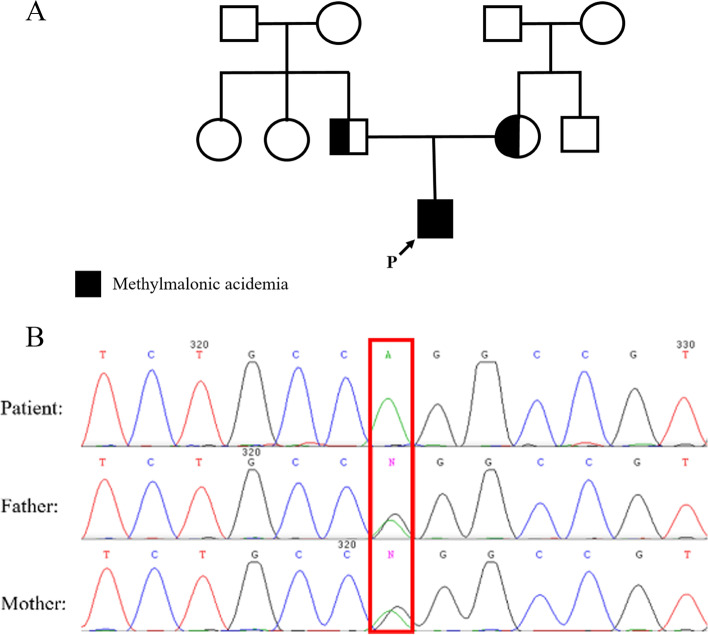
Pedigree and Sanger sequencing results. **A** Pedigree; **B** Sanger sequencing results show the homozygous and the heterozygous status of the proband and his parents, respectively

The proband was a 2-day-old neonate admitted to NICU for the symptoms of metabolic decompensation. His parents were not related (Fig. 1A), and in family history, no evidence of a known IMD was observed. The neonate presented vomiting, hypotonia, lethargy, respiratory distress, hypoglycemia, and metabolic acidosis in clinical examination. The neonatal reflexes, sucking, Moro, and palmar grasping, were abolished entirely. Biochemical tests revealed the following abnormalities: Blood Sugar < 20, Ammonia = 590, Lactate = 56, Bill Total = 9.5, Bill Direct = 0.5, AST = 260, ALT = 62, PT = 18.6, PTT = 56, Uric acid = 12.4, and Metabolic acidosis, suggesting a metabolic crisis. However, specific metabolic tests were unavailable, such as DBS or plasma acylcarnitine profiling, plasma amino acids profiling, urine organic acids measurement, and any other metabolic tests.

### Mutation analysis

In WES data analysis, following quality control, alignment, variant calling, and annotation, consecutive filtering steps were performed to narrow down the variants from 193,611 to 11 homozygous and 39 heterozygous variants. Both homozygous and heterozygous variants were considered, as the proband was a sporadic case in the family. The variants with minor allele frequency (MAF) < 0.05 were included in the last step of filtering to prevent the unexpected loss of any relevant variant. Moreover, this allows the analyzer to reanalyze the previous steps in case none of the final variants were incompatible with the clinical signs. The 11 homozygous and 39 heterozygous variants were subsequently assessed in gene datasets such as OMIM (https://www.omim.org/), GeneCards (https://www.genecards.org/), and GeneReviews (https://www.ncbi.nlm.nih.gov/books/NBK1116/); and disease datasets like ClinVar (https://www.ncbi.nlm.nih.gov/clinvar/), InterVar (https://wintervar.wglab.org/), and Varsome (https://varsome.com/) to compare the significance of variants. Finally, the variant most attributed to the clinical and biochemical presentations was a homozygous missense variant located on chromosome 12, NM_052845.4 (*MMAB*):c.557G > A (p.Arg186Gln), in exon 7 of the gene. Based on the ClinVar, InterVar, and Varsome datasets (2020), this variant was categorized into the variant with unknown significance (VUS) group of variants. Nevertheless, ClinVar’s submissions consist of one pathogenic, two likely-pathogenic, and one VUS (https://www.ncbi.nlm.nih.gov/clinvar/). This variant is responsible for isolated methylmalonic acidemia due to a defect in the synthesis of adenosylcobalamin, *cblB* complementation type, with an autosomal recessive mode of inheritance.

### Confirmatory analysis by Sanger sequencing

The final variant achieved in WES data analysis was successfully confirmed through Sanger sequencing in the affected neonate and his parents. As depicted in Fig. 1B, the neonate was homozygous, and his parents were heterozygous for this variant.

### Modeling and protein structure analysis

Although the pathogenicity of NM_052845.4 (*MMAB*):c.557G > A, p.Arg186Gln was uncertain, Uniprot (https://www.uniprot.org/uniprot/Q96EY8) and ClinVar (https://www.ncbi.nlm.nih.gov/clinvar/) datasets had reported the variant NM_052845.4 (*MMAB*):c.556C > T, p.Arg186Trp as pathogenic [5]. Hence, homology modeling and different bioinformatics tools were used to compare both variants’ structure and their effects on the protein function with the wild-type MMAB.

Homology modeling of MMAB protein by SWISS-MODEL resulted in the homo*-*trimer 3D structure using 6D5K-ABC as the template, where the sequence identity, sequence similarity, GMQE, and QMEANDisCo scores of the query to the template were 99.49%, 61%, 0.71 and 0.84, respectively. GMQE gives a quality estimate of the model based on the sequence coverage, and QMEANDisCo evaluates the reliability of the prediction based on model size. The scores are between 0 and 1, and higher scores show higher model quality.

**Fig. 2:**
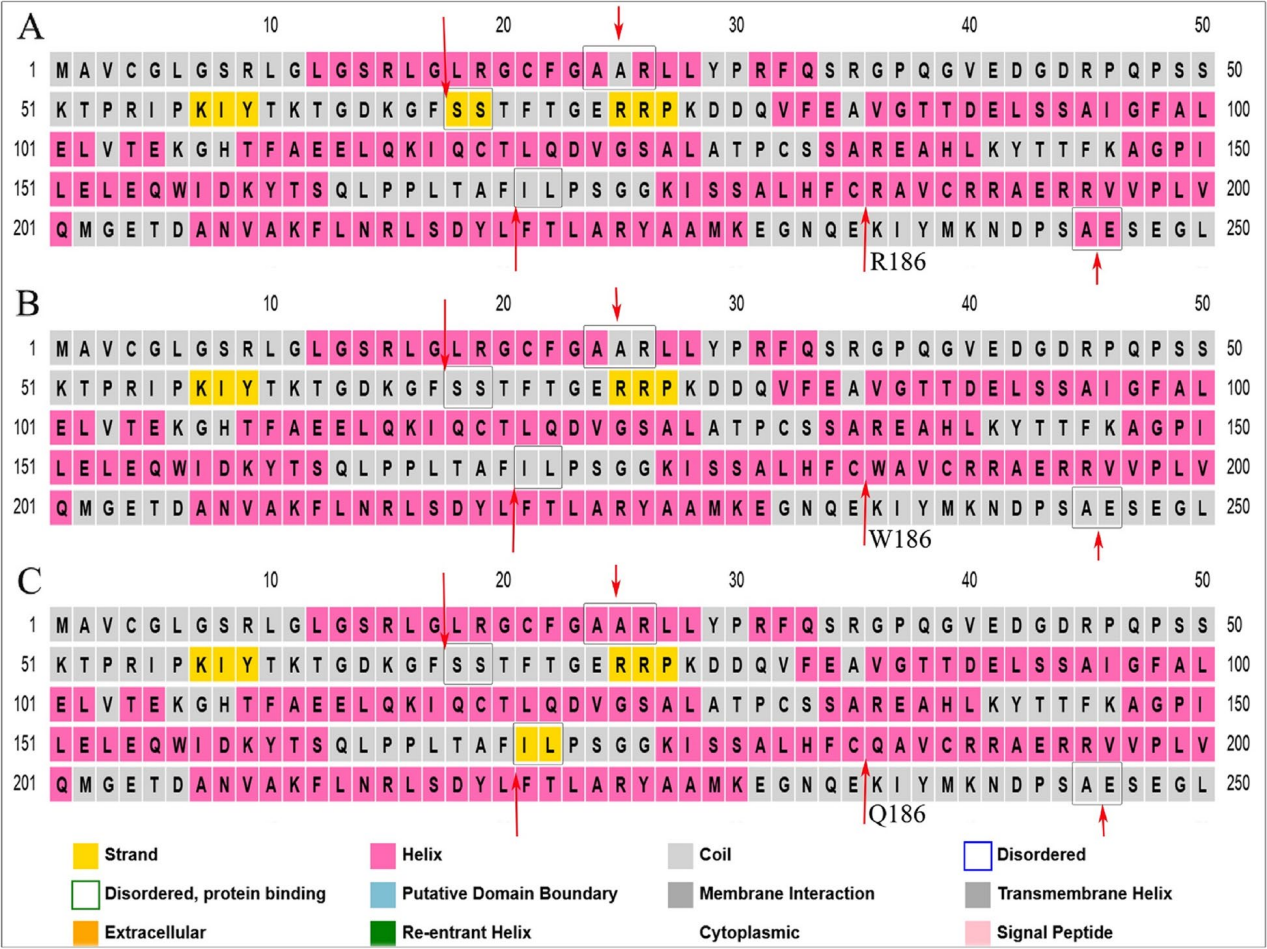
Prediction of the secondary structure of wild-type and MMAB variants. Secondary structure of wild-type (**A**), R186W (**B**), and R186Q (**C**) proteins prediction by PSIPRED 4.0

The sequence of wild-type MMAB and variants was submitted in the PSIPRED workbench to predict the secondary structure of proteins using PSIPRED 4.0. PSI-blast-based secondary structure PREDiction (PSIPRED) uses artificial neural network machine learning methods to assign regions of the sequence as a helix (H: pink), coil (C: gray), and strand (E: yellow). Despite no changes around residue 186 in the variants, there were some differences in the predicted results. For instance, while the predicted structure of residues 24–26 (AAR) was H-C-H in the wild-type, it was H-C–C and H–H-H in the R186W and R186Q variants, respectively. Likewise, residues 68–69 (SS) as nucleotide-binding sites (ATP) were in strand form in wild-type, though they were predicted as a coil in both variants. Moreover, residues 245–246 (AE) showed a helix structure in wild-type that had been expected as a coil in both variants. Furthermore, residues 171–172 (IL) in the R186Q variant were assigned as a strand similarly predicted as a coil in the wild-type and R186W variant (Fig. 2).

### The distinct binding pattern of wild-type and MMAB variants to ligands

**Fig. 3:**
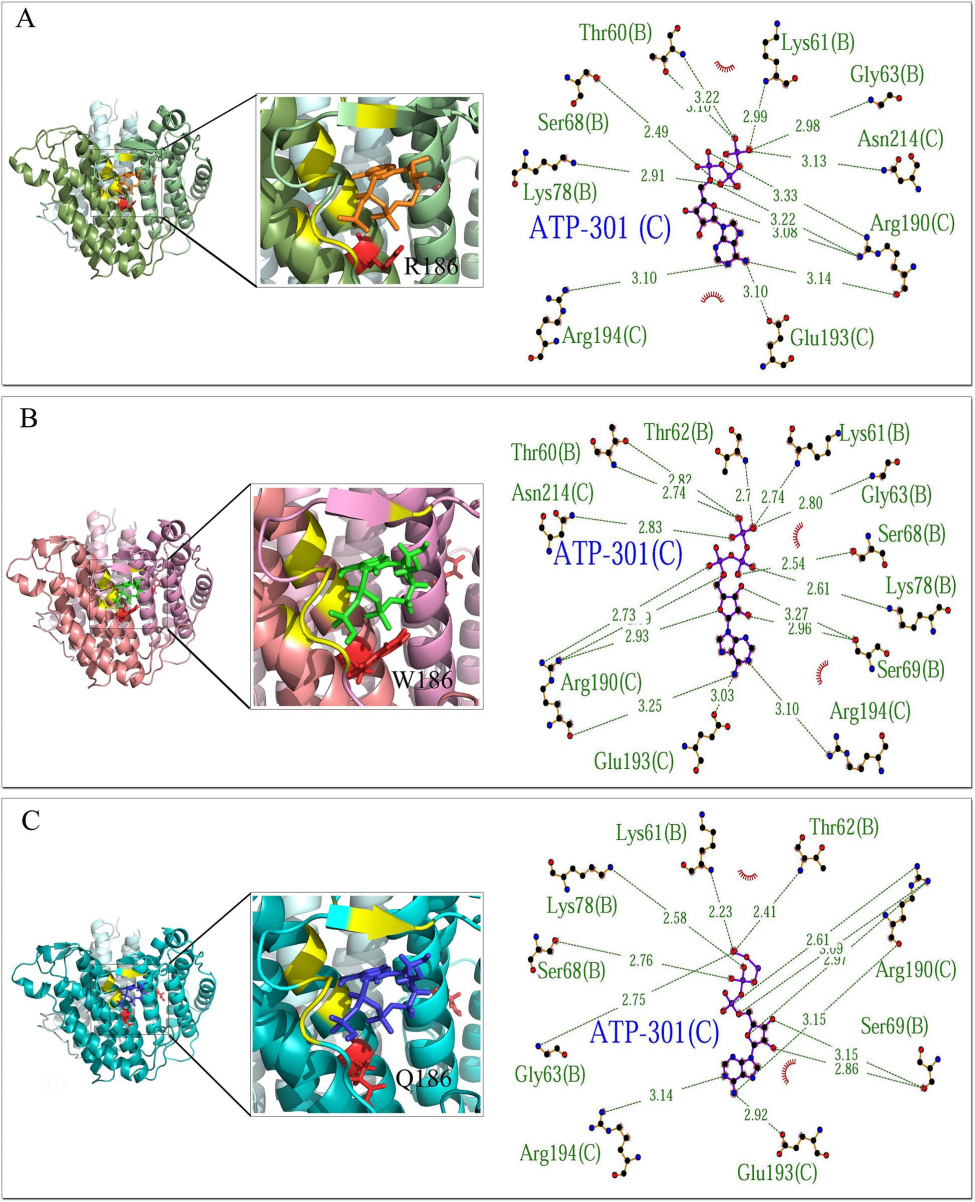
Prediction of the binding pattern of wild-type and variants MMAB to the ATP. The binding pattern of wild-type (**A**), R186W (**B**), and R186Q (**C**) proteins with the ATP

**Fig. 4:**
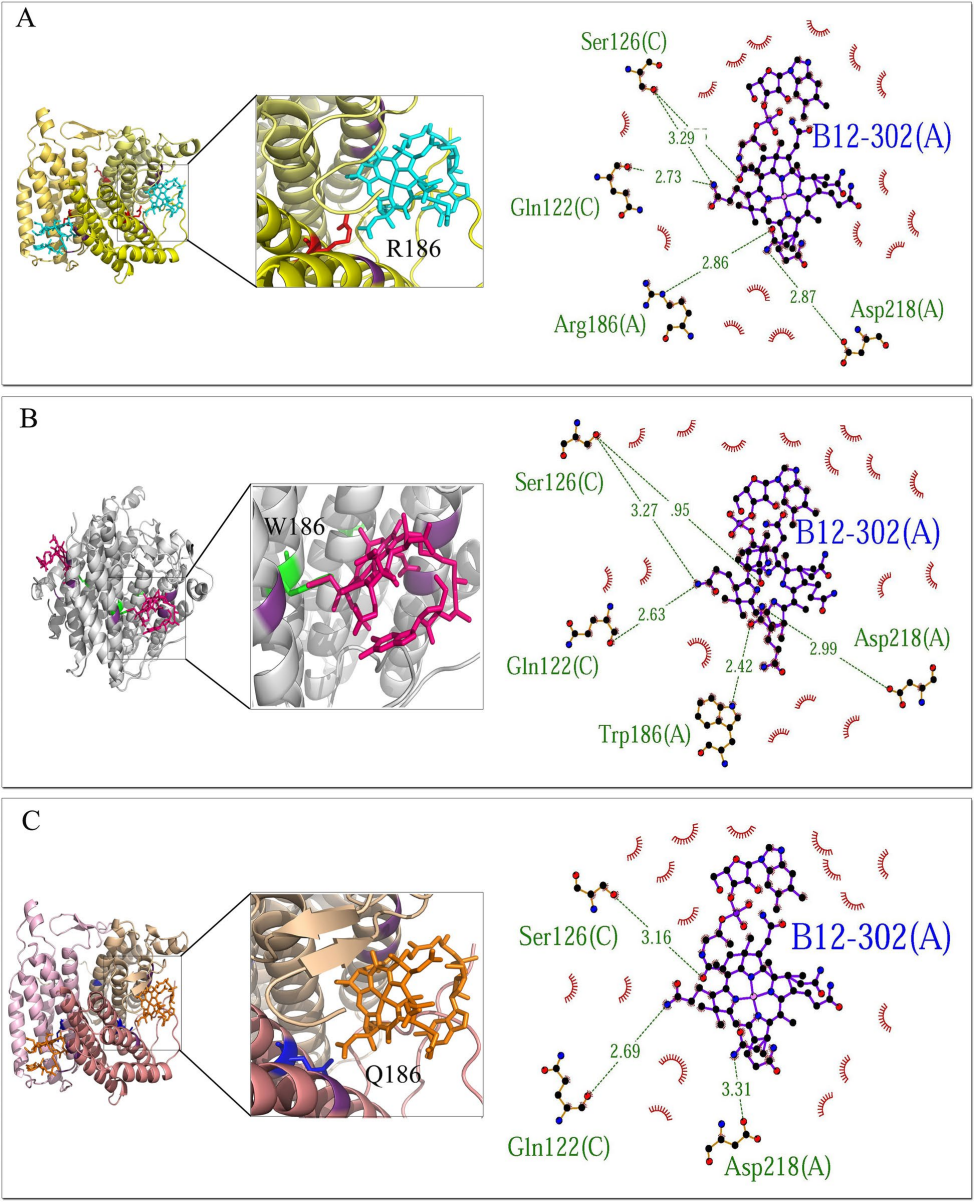
Prediction of the binding pattern of wild-type and variants MMAB to the B12-302. The binding pattern of wild-type (**A**), R186W (**B**), and R186Q (**C**) proteins with the B12-302 molecule

**Fig. 5:**
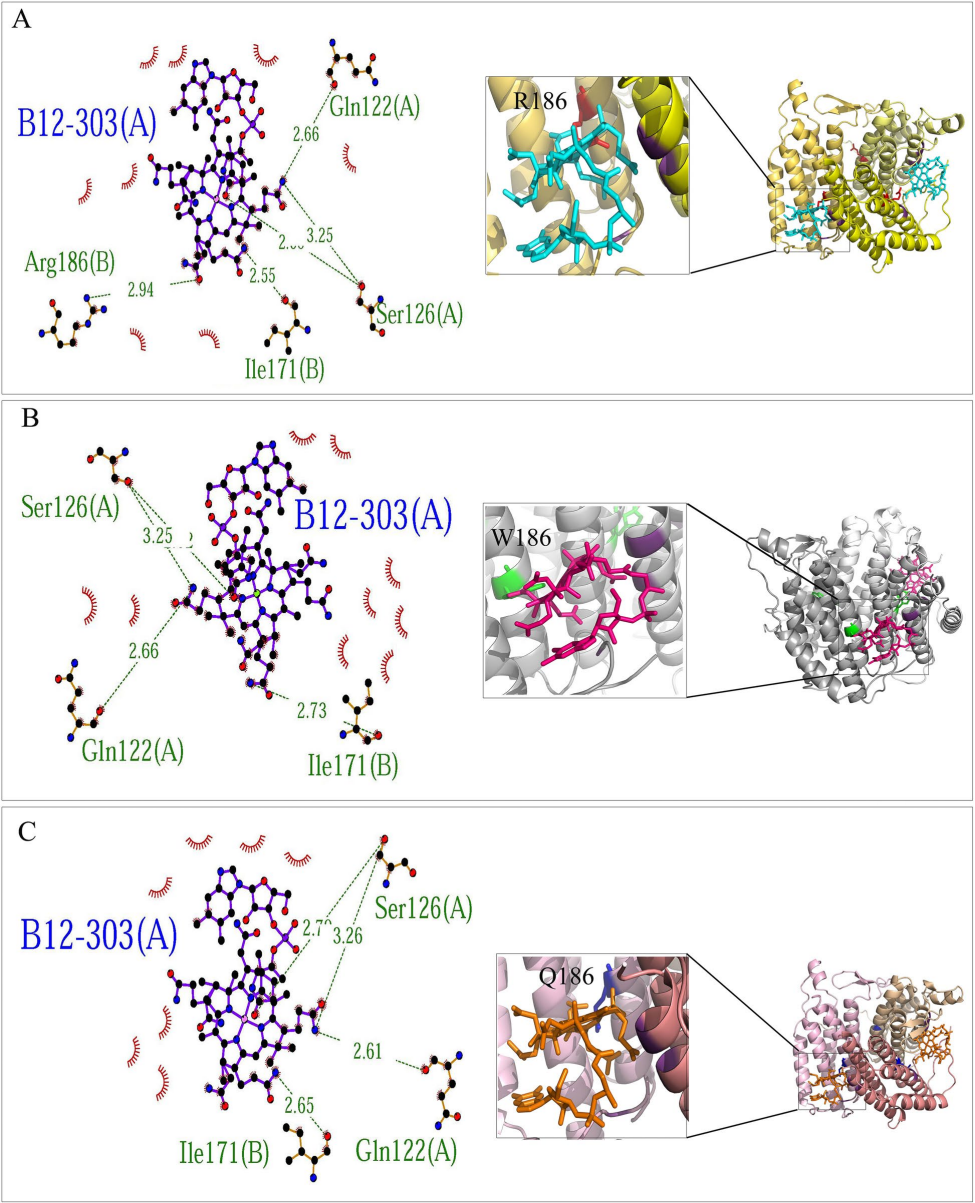
Prediction of the binding pattern of wild-type and variants MMAB to the B12-303. The binding pattern of wild-type (**A**), R186W (**B**), and R186Q (**C**) proteins with the B12-303 molecule

The model contains one ATP molecule (301) and two B12 molecules (302 and 303). LigPlot + extracted the interaction pattern of each ligand to protein in a 2D plot indicating hydrogen bonds between ligand and protein chains and the surrounding hydrophobic residues. ATP demonstrated interactions with chains B and C of MMAB protein. The common residues involved in hydrogen bonds to ATP included K61(B), G63 (B), S68 (B), K78 (B), R190 (C), E193 (C), and R194 (C). Despite the common residues of T60 (B) and N214 (C) in the interaction with ATP in both wild-type and R186W variants, these residues were not involved in R186Q/ATP complex. Also, R186Q and R186W variants formed extra hydrogen bonds to ATP with common residues T62 (B) and S69 (B) (Fig. 3). The B12-302 had lost the interaction with residue 186 (A) in the R186Q variant. However, it showed interactions with chains A and C, where residues R186 (A), D218 (A), Q122 (C), and S126 (C) were involved in the hydrogen bonds in wild-type protein, and W186 (A), D218 (A), Q122 (C), and S126 (C) in R186W variant (Fig. 4). B12-303 formed hydrogen bonds to residues Q122 (A), S126 (A), R186 (B), and I171 (B) in the wild-type protein. Residue 186 (B)’s interaction with B12-303 had been lost in both R186Q and R186W variants (Fig. 5). The cartoon view of 3D structures and 2D plots are depicted in Figs. 3, 4, and 5.

Finally, the PRODIGY server was used to calculate the binding affinity (ΔG) of ligands to protein.

As a result, protein–ligand binding affinity to ATP molecules showed no significant difference in wild-type and variants, consistent with a relatively similar binding pattern of protein residues (Table 1). Likewise, binding affinity to B12-302 was -8.1 to -8.2 kcal/mol, though B12-303 showed slightly higher binding tendency in R186Q and R186W than the wild-type residue (-8.3, -8.6, and -8.1 kcal/mol, respectively) (Table 1).

**Table 1:** Binding affinity and residues in hydrogen bonds to B12 and ATP ligands

Proteins	ΔG_noelec_ (Kcal/ mol)	Residues in H-bond	ΔG_noelec_ (Kcal/ mol)	Residues in H-bond	ΔG_noelec_ (Kcal/ mol)	Residues in H-bond
	**B12-302**		**B12-303**		**ATP**	
**MMAB**	-8.1	R186 (A), R218 (A), Q122 (C), S126 (C)	-8.1	Q122 (A), S126 (A), I171 (B), R186 (B)	-5.7	T60 (B), K61 (B), G63 (B), S68 (B), K78 (B), R190 (C), E193 (C), R194 (C), N214 (C)
**MMAB-R186Q**	-8.2	R218 (A), Q122 (C), S126 (C)	-8.3	Q122 (A), S126 (A), I171 (B)	-5.6	K61 (B), T62 (B), G63 (B), S68 (B), S69 (B), K78 (B), R190 (C), E193 (C), R194 (C)
**MMAB-R186W**	-8.2	W186 (A), R218 (A), Q122 (C), S126 (C)	-8.6	Q122 (A), S126 (A), I171 (B)	-5.6	T60 (B), K61 (B), T62 (B), G63 (B), S68(B), S69 (B), K78 (B), R190 (C), E193 (C), R194 (C), N214 (C)

### Analysis of phenotypic impacts by PROVEAN, SNAP2, and PANTHER cSNP

**Fig. 6:**
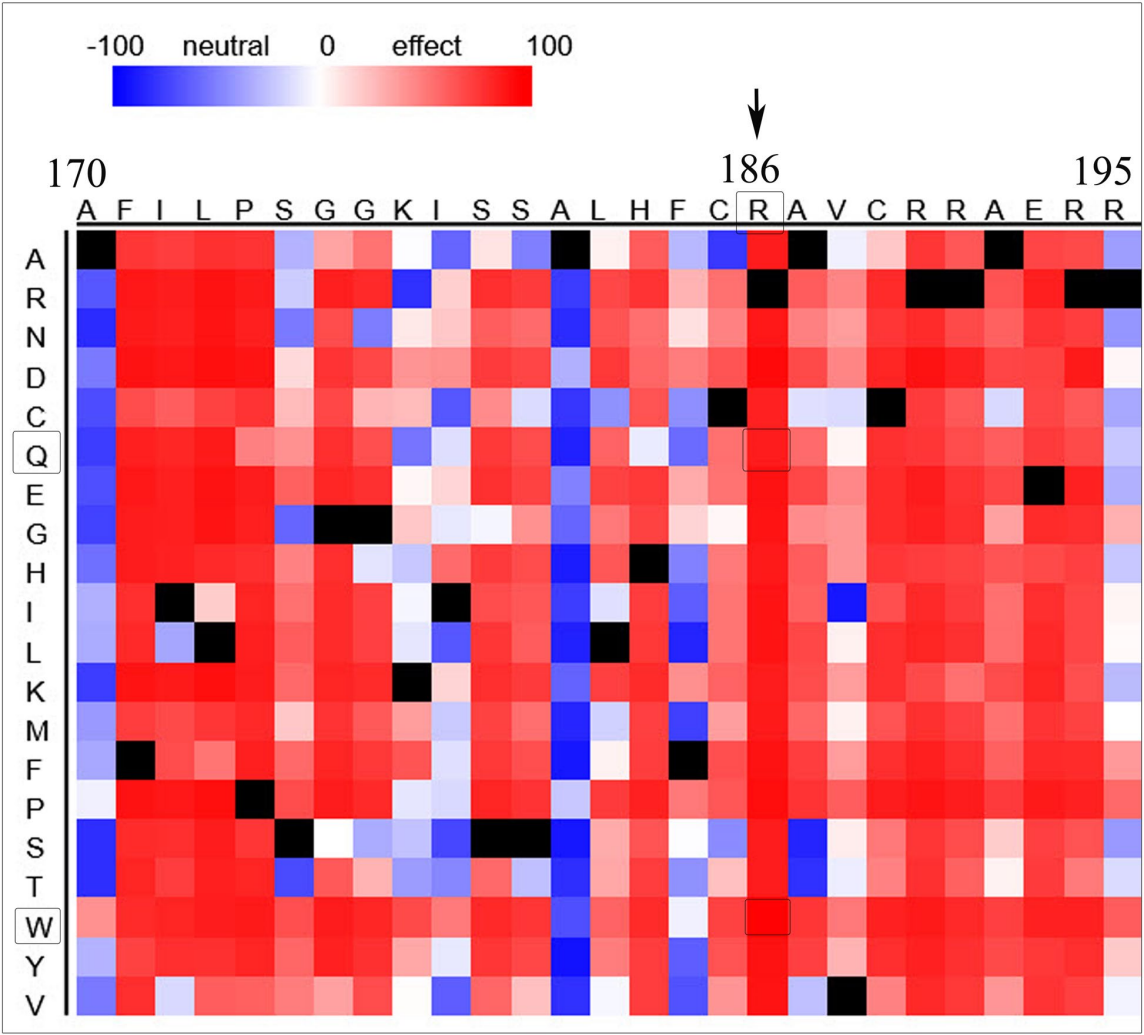
The heatmap representation of the functional effect for residues 155-210 of MMAB. The SNAP2 heatmap representation of the functional effect of amino acid substitutions for residues 155–210 of MMAB shows that substitution at position 186 to either W or Q strongly affects the protein function

PROVEAN scores indicated that both variants R186W and R186Q (scores of -7.46 and -3.7, respectively) were deleterious. Further, SNAP2, which prognosticates the impact of amino acid substitutions on protein function as a score ranging from -100 to + 100, from strong neutral to strong effect prediction, predicted R186W and R186Q as effective functional mutations with an expected accuracy of 95% and 91% and 99 and 89, respectively. The heatmap representation of the functional effect for residues 155–210 showed a strong impact of substitution at position 186 (Fig. 6). Likewise, the PANTHER-cSNPs tool evaluated the variants R186W and R186Q as probably damaging based on the evolutionary analysis of coding SNPs (Table 2).

**Table 2:** Evaluation of SNP effects on MMAB

**Variant**	**PROVEAN score**	**Prediction (cutoff = -2.5)**	**SNAP2 score**	**PANTHER**	**I-Mutant stability prediction**	**ΔΔG (Kcal/mol)**	**RI**^a^	**MUpro ΔΔG (Kcal/mol)**	**mCSM-NA ΔΔG (Kcal/mol)**
R186W	-7.46	Deleterious	99	damaging	decrease	-0.55	6	-0.717	-0.853
R186Q	-3.7	Deleterious	89	damaging	decrease	-1.09	8	-0.669	-1.255

^a^ Reliability Index

### Prediction of protein stability changes due to mutation effect using I-Mutant 2.0, Mupro, and mCSM-NA servers

The prediction of stability changes of MMAB due to R186W and R186Q variations by I-Mutant 2.0 showed a decrease in protein stability with the reliability index (RI) above 5. The values of ΔΔG for the variants R186W and R186Q (-0.55 and -1.09 kcal/mol, respectively) reflected the decrease of the free energy change upon amino acid substitution. Likewise, MUpro predicted the negative ΔΔG value for both variants, which means that both variants have decreased stability in protein structure. Also, the mCSM-NA server evaluated the effect of mutations as a destabilizing impact on proteins by the negative ΔΔG values for both variants (Table 2).

### Classification of the variant c.557G > A, p.Arg186Gln

Based on the ACMG guideline [11], gaining one strong, (PP3),^1^ two moderates, (PM1),^2^ and (PM5),^3^ and two supporting criteria, (PM2),^4^ and (PP5),^5^ the variant NM_052845.4 (*MMAB*):c.557G > A, p.Arg186Gln was categorized into the pathogenic group of variants [12].

### Haplotype analysis

**Fig. 7:**
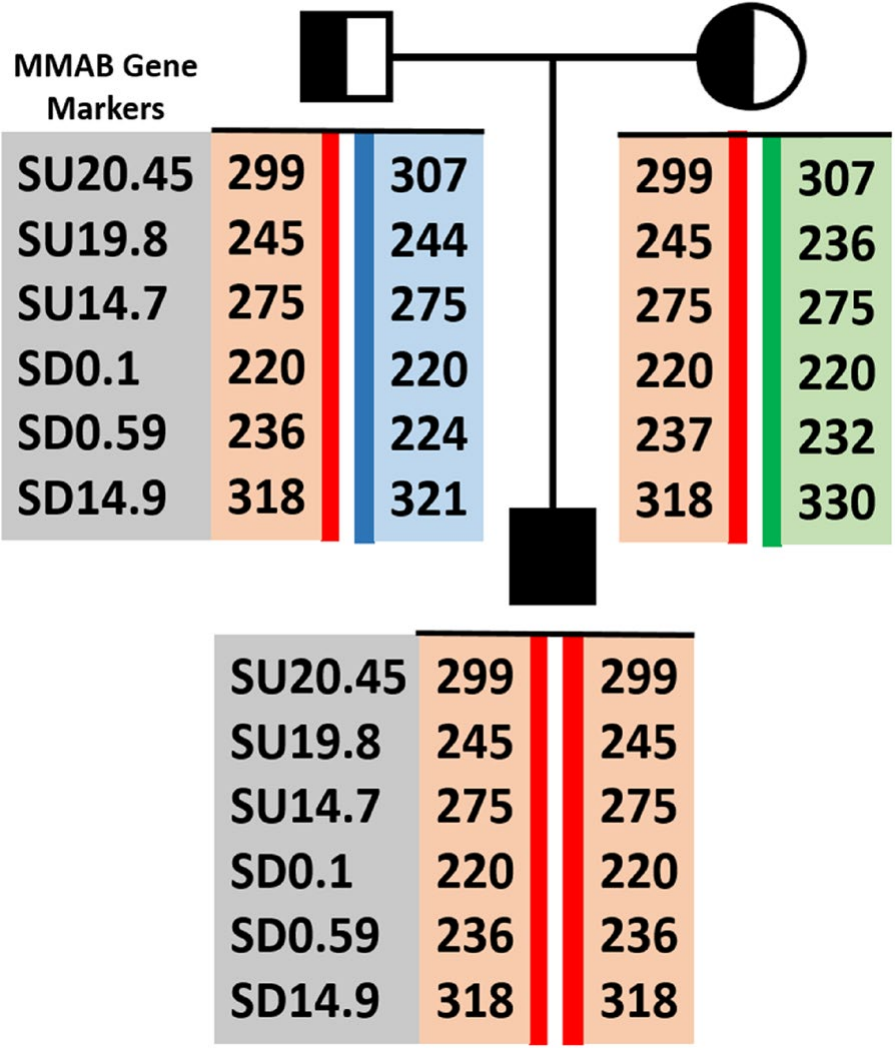
Haplotype mapping for the proband and his parents. SU20.45, SU19.8, and SU14.7 indicate upstream STRs, and SD0.1, SD0.59, and SD14.9 indicate downstream STRs of the *MMAB* gene

Evaluation of 6 different STRs, upstream (SU20.45, SU19.8, and SU14.7) and downstream (SD0.1, SD0.59, and SD14.9) of the *MMAB,* revealed that the parents share the same haplotype with their affected neonate (Fig. 7).

## Discussion

In the present study, we identified a single nucleotide variant in exon 7 of the *MMAB* gene, the NM_052845.4 (*MMAB*):c.557G > A, p.Arg186Gln, in WES data analysis of a neonate who died from an unknown metabolic disease. Evaluation of the effect of the variant on protein structure and function revealed some changes in the secondary structure and the protein affinity to the ligands, compatible with the changes in a known pathogenic variant at the same position. Based on the ACMG guideline, this variant was interpreted as pathogenic. Besides, the clinical signs and biochemical tests favored MMA, and the variant was heterozygous in the parents. Thus, the diagnosis was approved regarding WES results, confirmatory tests by Sanger sequencing, and in-silico protein structure and function evaluations.

MMA is an autosomal recessive organic acid metabolism disorder resulting from defects in MCM, an enzyme that converts methylmalonyl Co-A to succinyl Co-A, a substrate for the tricyclic acid cycle. This phenomenon leads to an increase in methylmalonic acid and other related metabolites in blood and urine. Mutations in various genes result in MMA, one of which is the *MMAB*, encoding ATR responsible for converting Cob(I)alamine to AdoCbl as the active form of the vitamin B12 (adenosyl cobalamine) and the cofactor of MCM [3, 5–8].

MMA follows an autosomal recessive mode of inheritance. In other words, two defective alleles are required for the disease’s occurrence. Hence, in countries with a high incidence of consanguineous marriages, like Iran (37.4%) [13], autosomal recessive disorders are more expected in related couples [14]. Interestingly, the neonate who died from MMA resulted from a non-consanguineous marriage; however, they both came from Saveh in the Markazi province in the center of Iran. Therefore, haplotype analysis successfully revealed that the parents share an identical haplotype with their affected son (Fig. 7), indicating having a common ancestor. Scrutinizing their originality showed that their ancestors all belonged to the nomads of the Shahsevan-e Baghdadi tribe, who was originally from Baghdad. Nader, the future king of the Afshar dynasty, moved this confederacy’s primary nucleus from Kirkuk’s suburbs to Khorassan in 1733. After the assassination of Nader Shah, they migrated to Fars province, and afterward, at the beginning of Qajar rule, they settled down in Qazvin, Hamedan, and Saveh, the birthplace of the proband’s parents [15].

Although the variant NM_052845.4 (*MMAB*):c.557G > A, p.Arg186Gln has been reported in the literature, its pathogenicity has not been accurately determined. Jordan P. Lerner-Ellis et al*.,* in 2006, for the first time, identified a homozygous form of this variant in a white boy whose diagnosis of *cblB* was established by somatic cell complementation analysis at the age of 14. No additional information was available on the clinical symptoms; however, the AdoCbl level in the cultured fibroblast cell line had been decreased to 2% (control = 15.3 ± 6.7%), and the Propionate uptake was diminished, too, indicating reduced MUT enzyme activity. They suggested the R186, accompanied by R190 and R191, as the putative enzyme’s active site and predicted this variant as a variant with uncertain significance (VUS) [16]. After the recent report of this variant [4], the present study is the third report and the first in-silico functional analysis of c.557G > A, p.Arg186Gln. However, functional studies on animal models would definitely approve the pathogenicity of this variant.

**Fig. 8:**
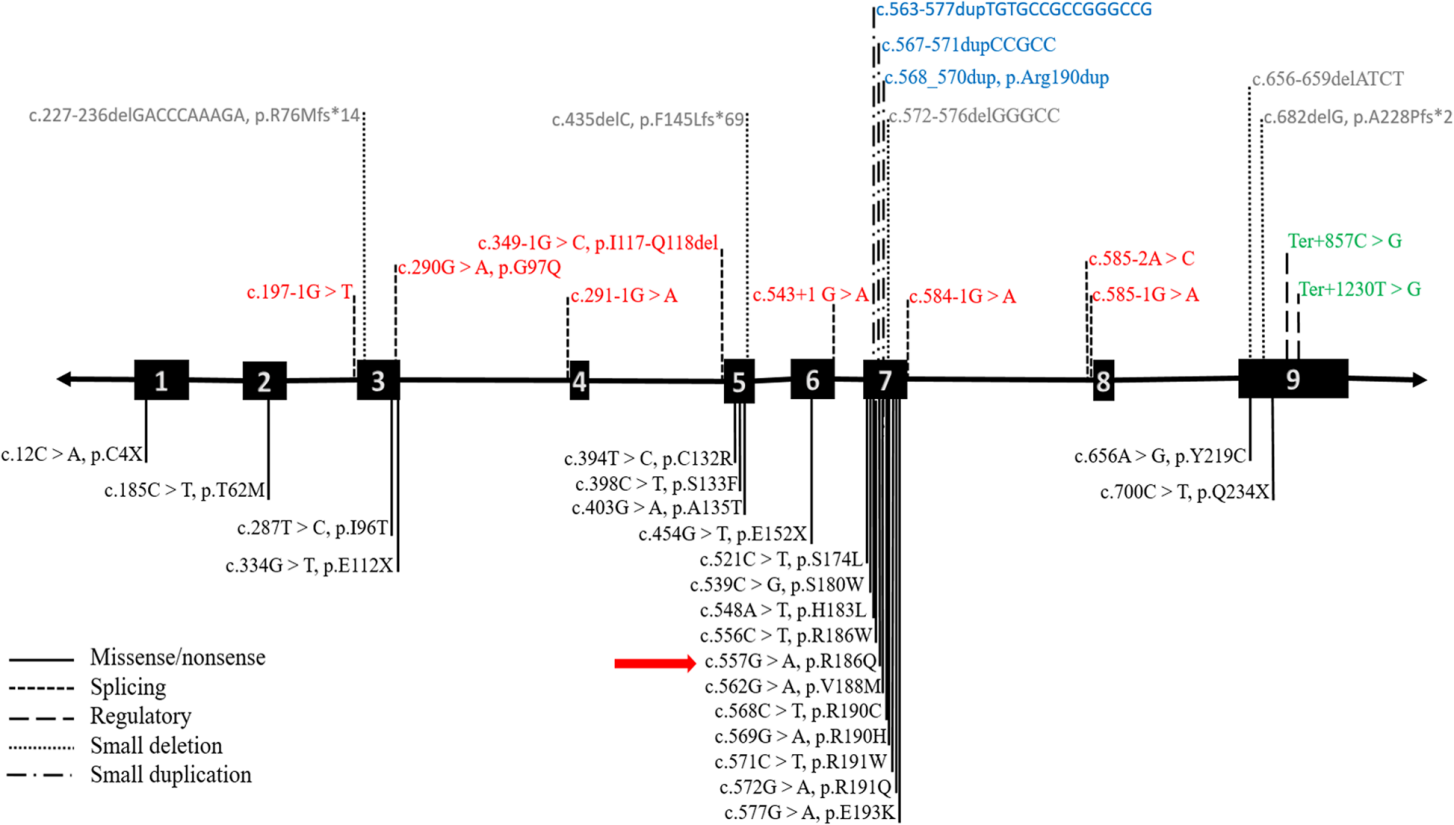
The mutation range of the *MMAB* gene. Each specific color demonstrates a type of variant. The black, red, gray, blue, and green colors indicate missense/nonsense, splicing, small deletion, small duplication, and regulatory variants, respectively. As shown, the majority of variants are aggregated on exon 7, indicating the existence of a hot spot in this exon

The remaining descriptions on position R186 belong to the NM_052845.4 (*MMAB*):c.556C > T, p.Arg186Trp, a known pathogenic variant with early-onset presentations [7, 16–18]. The mutation range of the *MMAB* gene is presented in Fig. 8 (https://www.hgmd.cf.ac.uk/). As depicted, most variants are clustered in exon 7, a small, highly conserved region of the *MMAB* gene, where both variants NM_052845.4 (*MMAB*):c.557G > A, p.Arg186Gln and c.556C > T, p.Arg186Trp are located.

The protein structure evaluations of the wild-type MMAB, p.R186W, and p.R186Q revealed residues 68–69 (SS), reported as nucleotide-binding sites for ATP molecule (https://www.uniprot.org/uniprot/Q96EY8), were changed from the strand form in Wild-type, to the coil in both variants. It suggests an alteration in the binding pattern to the ATP, the enhancer factor for the AdoCbl transfer to the MCM [3]. Likewise, both variants changed residues 245–246 (AE) from the helix format to the coil. Whereas residues 24–26 (AAR) showed various structures in the p.R186Q compared to p.R186W, both different from the wild-type protein. Noteworthy, residues 171–172 (IL), which showed no changes in the p.R186W, were modified to the strand form in the p.R186Q (Fig. 2).

Likewise, comparing the binding pattern of the proteins to the ATP, B12-302, and B12-303, in wild-type, p.R186W and p.R186Q demonstrated residues 62 (T) and 69 (S) from chain B of both variants were aberrantly involved in the hydrogen bond to the ATP. Furthermore, unlike p.R186W, the p.R186Q variant had lost the hydrogen bond to the ATP at positions 60 (T) chain B and 214 (N) chain C (Fig. 3). The same results were observed in the binding pattern of residue 186 of chain A with the B12-302 molecule, which lost the hydrogen bond in variant p.R186Q (Fig. 4). Moreover, residue 186 of chain B had lost the interaction with the B12-303 molecule in both variants (Fig. 5). In brief, according to the results obtained from in-silico evaluations of variant proteins’ structure and function, p.R186Q had more structural and functional variation than p.R186W; however, the functional analysis was not available.

## Conclusion

Considering the result of this study, the variant NM_052845.4 (*MMAB*):c.557G > A, p.Arg186Gln is suggested as a pathogenic variant and the cause of a severe early-onset form of MMA and neonatal death in the present case. These results could benefit the geneticists, specialists in the endocrinology and metabolism field, the patients, and their families who have undergone prenatal diagnosis. As the next step forward, Functional studies on animal models and studies with a larger sample size are suggested to evaluate the allele frequency of the present variation in the Iranian population. Furthermore, this approach could be beneficial in assessing the results of WES and the accuracy of variant pathogenicity interpretation and molecular diagnosis of genetic diseases.

## Methods

### Individuals

A 2-day-old neonate, suspected of an inherited metabolic disorder (IMD), was admitted to the NICU section of Ali-Asghar Pediatric Hospital, Iran University of Medical Science, Tehran, Iran. Following appropriate therapeutic approaches, pre-test genetic counseling was carried out, and the parents were given written informed consent for participation in this study and publication of the pertinent data included in this article. All steps of this study were settled regarding the National Ethical Standards. The project was authorized by the Ethics Committee of the Pasteur Institute of Iran, with the approval code of IR.PII.REC.1397.70.

### Sample collection and DNA extraction

DNA was extracted using QIAamp DNA Blood Mini Kit (QIAGEN, Hilden, Germany) from 5 ml of peripheral blood of the patient and his parents. The quality and quantity of DNA samples were assessed by Thermo Scientific™ NanoDrop™ (1.8 > A280 / A260 > 2) (Thermo Scientific, Waltham, MA, USA) as well as running on a 2% agarose gel.

### Whole-exome sequencing

Whole-Exome Sequencing (WES) was conducted to detect the disease’s genetic variant. For this purpose, SureSelect Human All Exon V6 kit (Agilent Technologies, Santa Clara, CA, USA) was utilized to enrich all exons of protein-coding genes and the flanking sequences of fragmented genomic DNA. Subsequently, the generated library was sequenced on the NextSeq 500 platform (Illumina, San Diego, CA, USA) to an average coverage depth of 100X. After the base calling, adapters trimming, and FASTQ file quality controls, the primary filtering stage was performed to eliminate the low-quality reads and probable artifacts. Afterward, reads alignment to the reference human genome (hg19) using Burrows-Wheeler Alignment (BWA) tool [19], duplication removal with Picard-Tools (http://broadinstitute.github.io/picard), and variant calling by HaplotypeCaller tool from Genome Analysis Tool kit version 4.0 (GATK4) package accomplished [20]. To annotate the variants, ANNOVAR was applied, extracting the allele frequencies from population databases, 1000 Genomes project (1000G) [21], the exome aggregation consortium (ExAC, https:// exac.broadinstitute.org/) [22], genome aggregation database (gnomAD, https:// gnomad.broadinstitute.org/) [23], and Kaviar dataset (https://db.systemsbiology.net/kaviar/) [24]. Probable effects of prioritized variants on protein structure and function were predicted utilizing open-access pathogenicity predictors, SIFT (https://sift.bii.a-star.edu.sg/) [25], PolyPhen-2 (http://genetics.bwh.harvard.edu/pph2/) [26], MutationTaster (http://www.mutationtaster.org/) [27], PANTHER (http://www.pantherdb.org/) [28], and PROVEAN (http://provean.jcvi.org/) algorithms [29].

The derived variants were filtered sequentially as the next step, based on prioritization criteria. The variants obtained from the final filtering stage were clinically and biochemically investigated in Online Mendelian Inheritance in Man (OMIM) (https://www.omim.org/), GeneCards (https://www.genecards.org/), and GeneReviews® (https://www.ncbi.nlm.nih.gov/books/NBK1116/) databases to exclude unrelated variants. Concomitantly, the final variants’ pathogenicity was evaluated in ClinVar (https://www.ncbi.nlm.nih.gov/clinvar/), InterVar (http://wintervar.wglab.org/), dbSNP (https://www.ncbi.nlm.nih.gov/snp/), and Varsome (https://varsome.com/variant/hg19/) datasets [12]. The ultimate variant was also reviewed in the in-house population database, Iranome (http://www.iranome.ir), to assess the allele frequency in Iranian ethnicity.

### Confirmatory analysis by Sanger sequencing

To confirm the variant attained from the filtering steps of WES analysis, appropriate forward and reverse primers were designed using GeneRunner software (version 5.1.6), NCBI (https://www.ncbi.nlm.nih.gov/), Ensembl Genome Browser (https://asia.ensembl.org/), NCBI-Nucleotide BLAST (https://blast.ncbi.nlm.nih.gov/Blast.cgi), and UCSC Genome Browser (https://genome.ucsc.edu/) databases, to amplify the desired gene fragment. The primer sequences are available on request.

The polymerase chain reaction (PCR) was performed in an Applied Biosystems (ABI) thermal cycler 2720 (Thermo Fisher Scientific, USA, TF), while each reaction contained ten ng of genomic DNA and KBC Master Mix (Kawsar Biotech Co., KBC, Tehran, Iran), following the producer protocol, in a final volume of 20 µl. The thermal cycling program consisted of an initial denaturation step at 94^ °C^, 4 min, followed by 28 cycles of PCR, including denaturation at 94^ °C^, 60 s, annealing at 63^ °C^, 60 s, and extension at 72^ °C^, 90 s, followed by the last extension step at 72^ °C^ for 10 min. PCR products were electrophoresed on a 1.5% agarose gel to ensure quality of PCR products.

Further, the products were sequenced using a BigDye Terminator version 3.1 (TF) on a 3130/xl Genetic Analyzer (Applied Biosystems, Carlsbad, CA). Afterward, Chromas software version 2.31 (Technelysium, Brisbane, Australia) was used to analyze the Sanger sequencing data. The sequences were aligned to the human reference sequence, version hg19 applying NCBI-Nucleotide BLAST (https://blast.ncbi.nlm.nih.gov/). Then, this variant’s pathogenicity was classified according to the American College of Medical Genetics and Genomics (ACMG) guidelines [11].

### Protein structure analysis

The structure and function of the Corrinoid adenosyltransferase enzyme, encoded by the *MMAB* gene, were evaluated in UniProt (https://www.uniprot.org/), RCSB PDB (https://www.rcsb.org/), and NCBI (https://www.ncbi.nlm.nih.gov/) servers to assess the effect of the amino acid substitution on protein structure and function. Protein residue conservations were also examined using the ConSurf server (http://consurf.tau.ac.il/2016/) [30].

### Modeling and protein structure analysis

The amino acid sequence of MMAB_human corrinoid adenosyltransferase was retrieved from the UniProtKB database (ID: Q96EY8) to investigate proper 3D structure using the SWISS-MODEL web server [31]. After sequence alignment of the query and templates, the homology modeling pipeline of the web server generated the models based on ProMod3. The model was considered by GMQE (Global Model Quality Estimation), QMEAN scores, and sequence identity to represent the reliability and quality. The homo-trimer crystal structure of the human corrinoid adenosyltransferase protein bound to ATP and two cobalamin molecules (B12) was the template in modeling (PDB ID: 6D5K) [32]. The human Corrinoid adenosyltransferase contains 250 amino acids, 196 of which (55–250) have been resolved in the crystal structure with a resolution of 2.50 Å. The homo-trimer mutated models (R186W and R186Q) have been built using Chimera (version 1.8) [33]. The amino acid substitution in each model was energy minimized using the steepest descent and conjugate gradient algorithms (step size = 0.02 Å) and AMBER f12SB force field parameters to eliminate the atomic clashes of the structure. Then, the secondary structure of MMAB and variants was predicted using PSIPRED 4.0 [34], and the 2D plot of ligand–protein interactions was obtained using LigPlot + software [35]. In the end, the 3D structures were visualized by PyMOL [36], and the binding affinity (ΔG) of ligands to protein was calculated using the PRODIGY server [37].

### Analysis of the functional consequence of SNPs

The functional effects of R186Q and R186W on MMAB protein were investigated using PROVEAN, SNAP2, and PANTHER cSNP web servers. Protein Variation Effect Analyzer (PROVEAN) (http://provean.jcvi.org/index.php) predicts the phenotypic and biological effects of an amino acid substitution or indel based on sequence homology and clustering of BLAST hits by defining 75% global sequence identity. Evaluation generates the PROVEAN score that the value equal to or below a predefined threshold (-2.5) means a “deleterious” effect, and the score above it predicts a “neutral” effect of the mutation on the protein function [38].

Furthermore, the screening for Non-acceptable Polymorphisms (SNAP2) server (https://rostlab.org/services/snap2web/) predicts the functional effects of SNPs based on multiple sequences alignment, secondary structure, and solvent accessibility of the protein using the neural network method [39]. The result introduces a visual heatmap and a table with the prediction columns (effect or neutral), SNAP2 score (ranges from − 100 strong neutral prediction to + 100 strong effect prediction), and the expected accuracy.

Additionally, the mutation effect on protein in PANTHER cSNP (Protein analysis through evolutionary relationship-coding SNPs) is calculated based on the alignment of evolutionarily related proteins in subPSEC (Substitution Position Specific evolutionary conservation) [40]. The result indicates whether a mutation has been damaging during protein evolution.

### Prediction of the mutation effect on protein stability

The stability changes of MMAB protein due to R186Q and R186W variants were investigated by I-Mutant 2.0, mCSM, and MUpro web servers. I-Mutant 2.0 (http://folding.biofold.org/i-mutant/i-mutant2.0.html) calculates the free energy change value (ΔΔG) of protein starting from the sequence or the structure of the protein. It uses a support vector machine-based (SVM) tool to compute the mutation influence on protein stability. A positive ΔΔG value indicates an increase, and a negative value determines a decrease in protein stability [41]. Whereas Mutation Cutoff Scanning Matrix (mCSM) web server (http://biosig.unimelb.edu.au/mcsm/stability) measures the effect of a single mutation on protein stability by a structure-based approach using atomic distance patterns. The protein stability is computed based on changes in ∆∆G value, where the negative value indicates destabilizing protein compared to the wild-type [42]. Besides, MUpro evaluates the protein stability changes for single amino acid mutations using a set of machine learning programs (http://mupro.proteomics.ics.uci.edu/). It computes the value of energy change (ΔΔG), where a negative score means the mutation has decreased the protein stability [43].

### Haplotype mapping

The proband resulted from a non-consanguineous marriage, so six different Short Tandem Repeat (STR)s around the *MMAB* gene were applied to analyze the haplotypes in the affected neonate and his parents. Among extra-genic STRs, SU20.45, SU19.8, and SU14.7 were located upstream, and SD0.1, SD0.59, and SD14.9 were situated downstream of the *MMAB*. STR primers were all designed at Kawsar Human Genetics Research Center, Tehran, Iran.

## Data Availability

For confidentiality reasons, data on WES and other results are not public but they are available as well as primer sequences from the corresponding author Dr. Sirous Zeinali on reasonable request.

## Abbreviations

IUMS: Iran University of Medical Sciences
MMA: Methylmalonic acidemia
WES: Whole-exome sequencing
MCM: Methylmalonyl-CoA mutase
cblB-MMA: CblB type of methylmalonic acidemia
Cbl: Cobalamin
MS: Methionine synthase
AdoCbl: Adenosylcobalamin
MeCbl: Methylcobalamin
ATR: Adenosyltransferase
NICU: Neonatal Intensive Care Unit
ACMG: American College of Medical Genetics and Genomics
IMD: Inherited metabolic disorder
BWA: Burrows-Wheeler Alignment
GATK4: Genome Analysis Tool kit version 4.0
1000G: 1000 Genomes project
ExAC: Exome aggregation consortium
gnomAD: Genome aggregation database
OMIM: Online Mendelian Inheritance in Man
PCR: Polymerase chain reaction
ABI: Applied Biosystems
PROVEAN: Protein Variation Effect Analyzer
SNAP2: Screening for Non-acceptable Polymorphisms
PANTHER cSNP: Protein analysis through evolutionary relationship—coding SNPs
subPSEC: Substitution Position Specific evolutionary conservation
SVM: Support vector machine-based
mCSM: Mutation Cutoff Scanning Matrix
STR: Short Tandem Repeat
MAF: Minor allele frequency
VUS: Variant with unknown significance
PSIPRED: PSI-blast-based secondary structure PREDiction
RI: Reliability index
PGL: Primary gene list
NC-RNA: Non-coding RNA

## Footnotes

1 MetaRNN = 0.996 (greater than 0.939) in favor of strong pathogenicity.

2 Located in a mutational hot spot and/or critical and well-established functional domain (e.g., active site of an enzyme) without benign variation.

3 Missense change at an amino acid residue where a different missense change (p.R186W) determined to be pathogenic before.

4 Absent from controls (or at extremely low frequency if recessive) in Exome Sequencing Project, 1000 Genomes Project, or Exome Aggregation Consortium.

5 ClinVar classifies this variant as VUS, 1 star associated with Vitamin B12-Responsive Methylmalonic Acidemia Type *cblB*, with 4 submissions (1 P, 2 LP and 1 VUS).
